# Arsenical Phosphate of Soda

**Published:** 1900-06-23

**Authors:** 


The Hospital

A Journal of

The Medical Sciences and Hospital Administration.

Vol. XXVIII.—No. 717.] Edited by Sir Henry Burdett, K.C.B., and Solomon C. Smith, M.D., M.R.C.P. [June 23, 1900.

### Arsenical Phosphate of Soda.

Few circumstances could be better calculated to create alarm among the very numerous silly people who are in the habit of physicking themselves than the application made on Friday in last week to Mr. Curtis Bennett, on behalf of the Paddington Vestry, for summonses against two druggists for selling a "laxative drug, known as effervescent phosphate of soda, which upon analysis was found to contain in one case three and a half grains, and in the other eight and three-quarter grains, of arsenic to the pound." Until evidence has been heard, and a decision given, it would, of course, be improper to discuss the particular cases; but there can be no hesitation in agreeing with the Medical Officer of Health for Paddington in the view that the alleged adulteration was calculated to be dangerous to life; and it is currently reported that the compound in question has been somewhat widely distributed. The dose of effervescent phosphate of soda is stated in the Pharmacopœia at from 60 to 120 grains for repeated administration, and from a quarter to half an ounce for a single administration; and it belongs to a class of medicines which people who are their own physicians are very apt to take by the tablespoonful, without any strict care as to weighing or measuring. It is obvious that, in the worse of the two cases referred to, supposing the statements made in Court to be well founded, half an ounce of the preparation would contain a fourth of a grain of arsenic; while the repeated administration of a smaller dose might quite easily occasion symptoms of a very serious character, from which the sufferer would in all probability seek relief by the continued use of his favourite "laxative." The "Acqua Tofana" of mediæval Italy is supposed, we believe, to have been a somewhat dilute arsenical solution, not strong enough to be suspected from the operation of any single dose, but quite strong enough to kill when continued day by day. The possible consequences of the consumption of the Paddington variety of soda phosphate are scarcely more disquieting than the consideration of the channel by which the arsenic can have been introduced, or of the circumstances which led to its presence being suspected and sought for. These questions, if they can be fully investigated, will probably throw a good deal of light upon what are called "commercial" methods of drug manufacture. The compilers of the Pharmacopœia do not appear to have had before their thoughts even the possibility of the adulteration of phosphate of soda by arsenic, or at all events, they make no suggestion as to tests by which its absence may be demonstrated; nor is it easy to conceive, if the official method of manufacture were followed, from what source the poison can have been derived. It may reasonably be feared that the Vespasianic principle, "Non olet," is extensively held among manufacturers, and that, provided some passable imitation of the desired product can be turned out, very little care is taken of any quality other than cheapness in the materials from which it is obtained.

About half a century ago an extraordinary panic was created in England by the disclosures of the late Mr. Accum and of Dr. Hassall with regard to the prevalence and the character of food adulteration; and among the popular books of the period was one which rejoiced in the gruesome title of "Death in the Pot." It would be rash to say that the panic produced much alteration in the ways of the delinquents chiefly concerned, but it certainly had the effect of rendering large classes of retailers more cautious than they previously had been, so that it was comparatively easy for purchasers to obtain a guarantee of the absence of particularly objectionable ingredients from their purchases. Nevertheless, whatever might be the nature of the wares, people were compelled to eat, and under the operation of this primary law of nature inquiries gradually became less strict, and guarantees were less inquired for. It would be a very salutary consequence if the hearing of the Paddington summonses, or of others like them (and, as the occasion must have arisen from the action of wholesale manufacturers, it is hardly probable that they will stand alone), should give rise to a suspicion of "Death in the Drug Bottle," or if, in other words, it should have some tendency to call attention to the enormous amount of injury to health which is brought about by the foolish and indiscriminate employment of purgatives. This employment is largely due, of course, to the trust reposed by ignorant credulity in the advertisements of quack medicines, a large proportion of which appeal to the imagination of consumers chiefly by means of a purgative action; but few physicians, and still fewer surgeons, can be for a moment doubtful of the amount of injury which is thus occasioned to the public. It would probably be a useful revelation to ordinary consumers, if it were possible to bring home to them the fact that a large proportion of the members of the medical profession, at the close of long lives of health and usefulness, would be able to say that, after leaving the nursery or the school, they had never swallowed a purgative in the whole course of their existences.

